# Long-Term Alteration of Reactive Oxygen Species Led to Multidrug Resistance in MCF-7 Cells

**DOI:** 10.1155/2016/7053451

**Published:** 2016-12-12

**Authors:** Juan Cen, Li Zhang, Fangfang Liu, Feng Zhang, Bian-Sheng Ji

**Affiliations:** ^1^Key Laboratory of Natural Medicine and Immune Engineering, Henan University, Kaifeng, China; ^2^School of Pharmacy, Henan University, Kaifeng, China

## Abstract

Reactive oxygen species (ROS) play an important role in multidrug resistance (MDR). This study aimed to investigate the effects of long-term ROS alteration on MDR in MCF-7 cells and to explore its underlying mechanism. Our study showed both long-term treatments of H_2_O_2_ and glutathione (GSH) led to MDR with suppressed iROS levels in MCF-7 cells. Moreover, the MDR cells induced by 0.1 *μ*M H_2_O_2_ treatment for 20 weeks (MCF-7/ROS cells) had a higher viability and proliferative ability than the control MCF-7 cells. MCF-7/ROS cells also showed higher activity or content of intracellular antioxidants like glutathione peroxidase (GPx), GSH, superoxide dismutase (SOD), and catalase (CAT). Importantly, MCF-7/ROS cells were characterized by overexpression of MDR-related protein 1 (MRP1) and P-glycoprotein (P-gp), as well as their regulators NF-E2-related factor 2 (Nrf2), hypoxia-inducible factor 1 (HIF-1*α*), and the activation of PI3K/Akt pathway in upstream. Moreover, several typical MDR mediators, including glutathione S-transferase-*π* (GST-*π*) and c-Myc and Protein Kinase C*α* (PKC*α*), were also found to be upregulated in MCF-7/ROS cells. Collectively, our results suggest that ROS may be critical in the generation of MDR, which may provide new insights into understanding of mechanisms of MDR.

## 1. Introduction

Reactive oxygen species (ROS) in cells comprise a series of free radical molecules, which mainly includes superoxide anion (O_2_^∙−^), hydroxyl free radical (^∙^OH), singlet oxygen (^1^O_2_), and hydrogen peroxide (H_2_O_2_) [1]. ROS are normally generated by NAD(P)H oxidase isoforms, and intracellular antioxidants maintain the cellular redox homeostasis under physiological conditions [2], while, under pathological conditions, this balance is broken by the ROS enhancement, also known as the oxidative stress. Therefore, the intracellular ROS (iROS) level reaches a higher level and achieves a new equilibrium [3].

It has been well established that chemotherapy and radiation therapy eliminated tumor cells by ROS generation, because ROS were proposed as common mediators in the process of cell apoptosis [4]. However, ROS may not just act as the toxic by-products of metabolism, and recent studies suggested that ROS function as important second messengers and exert their functions by passive diffusion, water channel, and even signal waves [5]. In tumor cells, sustained production of ROS mainly activates survival signaling pathways such as PI3K/Akt and NF-*κ*B pathways, which facilitate oncogenic phenotype of cancer [6]. The targets of ROS also include growth factors, inflammatory factors, and cell cycle regulators, including c-Myc, NF-*κ*B, NF-E2-related factor 2 (Nrf2), hypoxia-inducible factor 1 (HIF-1*α*), and Protein Kinase C (PKC), and some of these factors are closely associated with multidrug resistance (MDR) in tumor [7]. Therefore, long-term exposure to sublethal ROS may account for the generation of MDR in cancer cells.

It has been well studied that ROS can induce antibiotic resistance in bacteria with enhanced multidrug efflux system [8]. Moreover, cancer cells were reported to have higher iROS compared with normal cells [9], and clinically, malignant tumor had higher environmental ROS (eROS) than benign tumor [10]. Therefore, we speculate that the iROS or eROS of MDR cells may also be different from those of their parental sensitive cells, and the differences may be critical for MDR generation. However, the generation of MDR is usually a complex outcome of clinical therapy, and ROS were rarely utilized as independent factors to study their relationship with MDR. Various studies have been using short-term (within days) oxidative stimulation to study the effect of ROS on MDR, but these findings were inconsistent and controversial [11, 12]. Therefore, we developed cell models with relative steady-state ROS by long-term incubation of oxidant or antioxidant, to study the effect of long-term ROS alteration on MDR. This study, for the first time, established the MDR cancer cell model by using human breast cancer MCF-7 cells, which were treated with low concentration of H_2_O_2_ and glutathione (GSH) for 20 weeks. The potential mechanisms and related signaling pathways were also investigated, which may provide reliable references for insights into mechanisms of MDR in cancer cells.

## 2. Materials and Methods

### 2.1. Materials

Fetal bovine serum (FBS) was obtained from Zhejiang Tianhang Biotechnology (Huzhou, China). 5-Diphenyl tetrazolium bromide (MTT), GSH, sulforhodamine B (SRB), rhodamine 123 (Rh123), Adriamycin (ADM), taxol, LY294002, and 3-(5′-hydroxymethyl-2′-furyl)-1-benzylindazole (YC-1) were purchased from Sigma Chemical (St. Louis, MO, USA). Dihydroethidium (DHE) was obtained from Molecular Probes (Invitrogen, Carlsbad, CA, USA). Polyclonal antibodies against MDR-related protein 1 (MRP1, sc-13960), P-glycoprotein (P-gp, sc-55510), Nrf2 (sc-722), NF-*κ*B-p-65(sc-372), HIF-1*α* (sc-10790), PKC*α* (sc-208), c-Myc (sc-789), glutathione S-transferase-*π* (GST*π*, ab53943), phosphatase and tensin homolog deleted on chromosome ten (PTEN, sc-7974), PI3K (ab182651), Akt (sc-8312), p-Akt (sc-7985-R) and *β*-actin (ab8227), and horseradish peroxidase-conjugated (BA1054, BA1050) or FITC-conjugated secondary antibodies (BA1101, BA1105) were purchased from Santa Cruz Biotechnology (sc-, CA, USA), Abcam (ab, MA, USA), and Boster Bio-Engineering Limited Company (BA, Wuhan, China). SiRNA kit for Nrf2 (Si-Nrf2) was from GenePharma Biotech Company (Shanghai, China). Whole cell lysis buffer containing proteasome inhibitor and BCA protein kit were products from Beyotime Institute of Biotechnology (Shanghai, China), and enhanced chemiluminescence (ECL) detection kit was obtained from Amersham Biosciences (Buckinghamshire, UK).

### 2.2. Cell Culture and Drug Treatment

**Figure 1:**
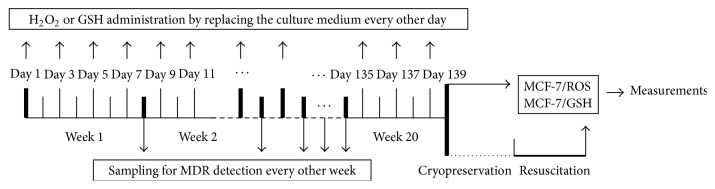
Scheme imaging course of the experiments.

MCF-7 cells were from American Type Culture Collection (ATCC, Manassas, VA, USA). MCF-7/ADM cells (KeyGen Biotech, Nanjing, China) were obtained from MCF-7 cells which were exposed to ADM with stepwise increased concentrations. To establish MDR cell models, MCF-7 cells were treated by replacing the culture medium (RPMI 1640 medium containing 10% FBS) every other day for 20 weeks. The culture medium, which contains H_2_O_2_ or GSH, was freshly prepared half an hour before medium exchange. The final concentrations were 0.001, 0.01, 0.1, 1, and 10 *μ*M for H_2_O_2_ groups and 0.001, 0.01, 0.1, 1, and 10 mM for GSH groups, respectively. Cells from negative control group were exposed to the same volume of PBS. Cells from positive control group were treated with 0.1 *μ*M ADM every other day for 20 weeks (namely, ADM group). In another ADM-treated group, 2 mM GSH was coadded for ROS abolishment (namely, ADM + GSH group). Forty-eight hours after the last administration, cells from each group were replaced with normal culture media without oxidant or antioxidant. Another forty-eight hours later, cells were harvested for further experiments (see sections below). Cells treated with 0.1 *μ*M H_2_O_2_ for 20 weeks were named as MCF-7/ROS, while cells treated with 0.1 mM GSH were named as MCF-7/GSH. Moreover, specific PI3K inhibitor LY294002 (10 *μ*M) [13], specific HIF-1*α* inhibitor YC-1 (5 *μ*M) [14], and Si-Nrf2 were applied on MCF-7/ROS cells for 48 h to inhibit PI3K, HIF-1*α*, and Nrf2, respectively. Scheme imaging course of the modeling process was shown in Figure 1.

### 2.3. MTT, SRB Assay, and 5-Bromo-2-deoxyuridine (BrdU) Incorporating Assay

Cells of each group were harvested and seeded in 96-well plates at a density of 1 × 10^5^ cells/mL for viability assay or 1 × 10^4^ cells/mL for proliferation assay. For the viability assay, cells were treated with ADM (1, 5, 25, 125, and 500 *μ*M) or taxol (1, 5, 25, 125, and 250 *μ*M) for 48 h, and then cells were incubated with MTT at 37°C for 4 h, and then the medium was removed and 150 *μ*L DMSO was added to each well. Plates were agitated and the optical density was measured at 570 nm using a spectrophotometer (Thermo Fisher Scientific Inc.).

**Figure 2:**
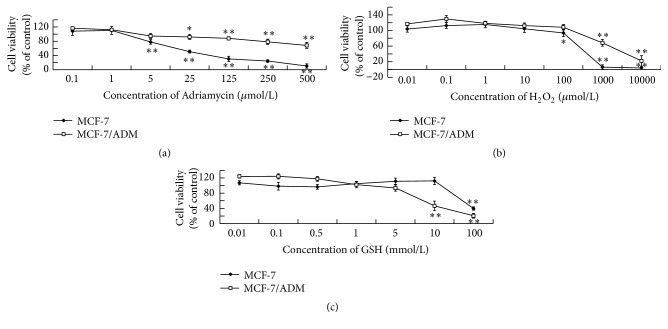
The effects of ADM, H_2_O_2_, and GSH on the viability of MCF-7 and MCF-7/ADM cells. Two days after the treatment, the effects of (a) ADM, (b) H_2_O_2_, and (c) GSH on the viability of MCF-7 and MCF-7/ADM cells were measured by SRB assay. Data represents the mean ± SD, *n* = 3, and significant differences of inhibition relative to control group were indicated as ^*∗*^ *P* < 0.05 and ^*∗∗*^ *P* < 0.01.

**Figure 3:**
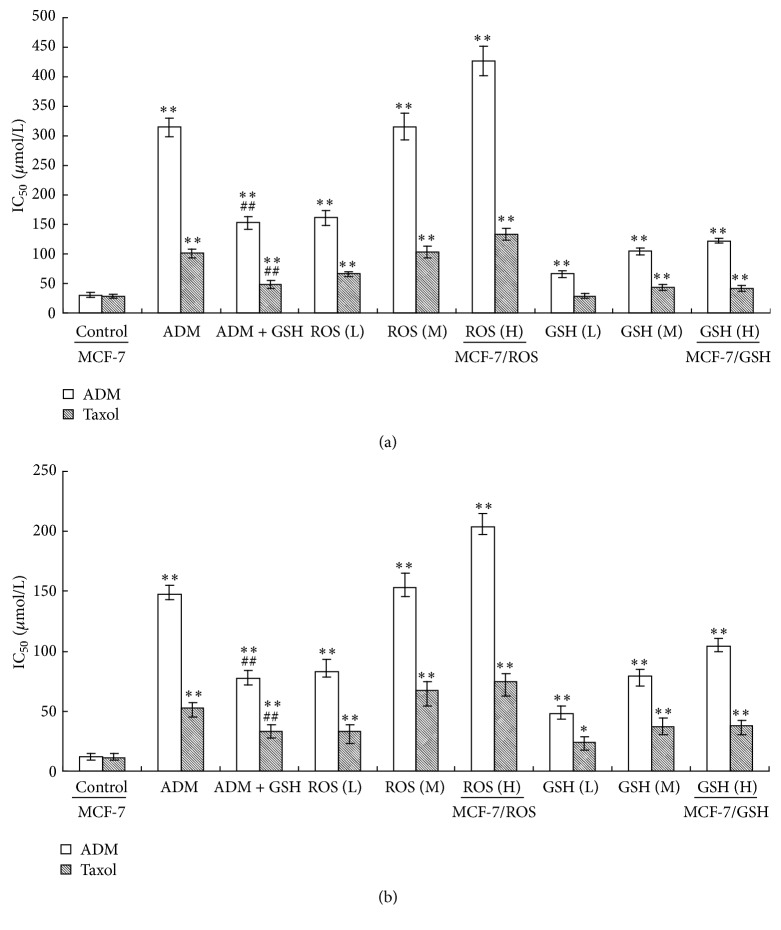
Long-term treatment of H_2_O_2_ and GSH induced MDR in MCF-7 cells. MCF-7 cells were treated by replacing the culture medium every other day for 20 weeks. MDR to ADM or taxol was determined by MTT (a) and SBR (b) assays. Control: normal culture medium (i.e., MCF-7 cells); ADM: 0.1 *μ*M ADM; ADM + GSH: 0.1 *μ*M ADM + 2 mM GSH; ROS (L): 0.001 *μ*M H_2_O_2_; ROS (M): 0.01 *μ*M H_2_O_2_; ROS (H): 0.1 *μ*M H_2_O_2_ (i.e., MCF-7/ROS cells); GSH (L): 0.001 *μ*M GSH; GSH (M): 0.01 *μ*M GSH; GSH (H): 0.1 *μ*M GSH (i.e., MCF-7/GSH cells). Data represents the mean ± SD, *n* = 3. Significant differences relative to control group were indicated as ^*∗*^ *P* < 0.05 and ^*∗∗*^ *P* < 0.01, and significant differences relative to ADM group were indicated as ^#^ *P* < 0.05 and ^##^ *P* < 0.01.

The SRB assay was performed by the method of Skehan et al. [15]. For the viability assay in Figure 2, cells were treated with ADM (0.1, 1, 5, 25, 125, 250, and 500 *μ*M), H_2_O_2_ (0.01, 0.1, 1, 10, 100, 1000, and 10000 *μ*M), GSH (0.01, 0.1, 1, 5, 10, and 100 mM), or taxol (1, 5, 25, 125, and 250 *μ*M) for 48 h. For the viability assay in Figure 3, cells were treated with normal culture medium for 48 h. The optical density was measured at 550 nm using a spectrophotometer (Thermo Fisher Scientific Inc.).

For cell proliferation analysis, a BrdU incorporation assay was performed using the BrdU cell proliferation assay kit (Cell Signaling Technology, Danvers, MA, USA). According to the manufacturer's instructions, absorbance was measured with a spectrophotometer at 450 nm.

Cell viability, proliferation rates, and inhibition rates were calculated on a plate-by-plate basis for test wells relative to control wells. IC_50_ was taken at the concentration that produced 50% inhibition of cell viability and was calculated from the inhibitory rate curves using Bliss' method. The resistance index (RI) was calculated by dividing IC_50_ of the MDR cells by IC_50_ of the respective non-MDR cells.

### 2.4. Flow Cytometric Analysis

Accumulation of Rh123 and ADM was determined by incubating cells with Rh123 (2 *μ*M) or ADM (5 *μ*M) for 1 h at 37°C. Cells were then placed in ice-water bath and followed by harvesting and washing twice with ice-cold PBS. The fluorescence intensity was measured to determine the intracellular drug accumulation.

### 2.5. DHE-Based ROS Detection by HPLC Analysis

MCF-7, MCF-7/ADM, MCF-7/ROS, and MCF-7/GSH cells were maintained in PBS and incubated with 50 *μ*M DHE for 30 min and then harvested to analyze DHE-derived products by HPLC [16]. Briefly, cells were washed twice with cold PBS, harvested in acetonitrile (0.5 mL/well), sonicated (10 s, 1 cycle at 8 W), and centrifuged (12,000 ×g for 10 min at 4°C). All extractions were performed with acetonitrile. Supernatants were dried and resuspended in 100 *μ*L PBS/DTPA and injected into HPLC system. Separation of DHE, EOH, and ethidium was performed as described [17] with specified modifications. Chromatographic separation was carried out with the use of a NovaPak C18 column (3.9 × 150 mm, 5 *μ*m particle size) in a HPLC system (Waters) equipped with a Rheodyne injector and photodiode array (W2996) and fluorescence (W2475) detectors. DHE was monitored by ultraviolet absorption at 245 nm. EOH and ethidium were monitored by fluorescence detection with excitation 510 nm and emission 595 nm. DHE-derived products were expressed as ratios of generated EOH and ethidium over consumed DHE.

### 2.6. Determination of Glutathione Peroxidase (GPx), GSH, Superoxide Dismutase (SOD), and Catalase (CAT)

After the cell collection, the medium was removed and the cells were washed thrice with PBS. Cells were dissociated by cell lysis buffer, and cell lysis was carried out at 4°C by vigorous shaking for 45 min. After centrifugation at 12000 rpm for 10 min, supernatant was separated and used to measure the GSH content, GPx, SOD, and CAT activities using assay kits based on the specified manufacturer's instructions (Jiancheng Institute of Biotechnology, Nanjing, China).

### 2.7. Immunofluorescence Staining

After fixation and permeabilization, cells were incubated with primary antibodies (1 : 50) against MRP1, P-gp, Nrf2, NF-*κ*B-p-65, and HIF-1*α* for overnight at 4°C. After washing with PBS twice, cells were incubated with FITC-labeled secondary antibodies (1 : 50) for 30 mins and then incubated with 10 *μ*g/mL DAPI and incubated for another 30 min. Signals were visualized and recorded using Confocal Microscopy at magnification of 400x.

### 2.8. Nrf2 siRNA Transfection and Quantitative Real-Time PCR (qRT-PCR)

Cells were transfected with Si-Nrf2 or control siRNA by using Lipofectamine™ 2000 reagent (Invitrogen, Carlsbad, CA) according to the manufacture's protocol. 48 hours after transfection, the mRNA extraction and qRT-PCR were carried out according to previous method [18]. Briefly, the total mRNA was extracted using TRIzol reagent (Invitrogen). Then Transcriptor First Strand cDNA synthesis kit (Takara, Takara Bio Inc., Otsu, Shiga, Japan) was applied to generate cDNA. The primers for human Nrf2 are 5′-TCAGCGACGGAAAGAGTATGA-3′ (forward primer) and 5′-CCACTGGTTTCTGACTGGATGT-3′ (reverse primer). The primers for human GAPDH are 5′-CGGAGTCAACGGATTTGGTCGTAT-3′ (forward primer) and 5′-AGCCTTCTCCATGGTGGTGAAGAC-3′ (reverse primer). The PCR was performed as follows: one cycle of denaturation (95°C for 30 s), 50 cycles of amplification (95°C for 5 s and 60°C for 34 s), and an annealing step of 60°C for 60 s. The level of mRNA was normalized to GAPDH.

### 2.9. Western Blot

Cells from each group were collected and suspended in lysis buffer for 30 min with shaking at 4°C. After centrifugation (10 000 ×g) for 10 mins, the supernatants were collected. Cell lysate (80 *μ*g) was resolved on 4–12% SDS-PAGE gels and then transferred on to nitrocellulose membranes. The membranes were blocked with tris-buffered saline with 0.1% Tween 20 and 5% skim milk and then incubated with primary antibodies (1 : 100–1 : 5000) overnight at 4°C and then washed with TBST for three times and incubated with HRP-conjugated secondary antibody (1 : 2000) at room temperature for 1 h. Following three-time wash with TBST, the membranes were developed by the ECL detection kit [19]. The images of western blot were captured and analyzed by Bio-Rad imaging system.

### 2.10. Statistical Analysis

All the experiments were performed in triplicate. All data were presented as means ± SD Significant differences between the groups were determined by the unpaired Student's *t*-test or one-way ANOVA followed by Dunnett's multiple comparison tests. *P* values less than 0.05 were considered as statistically significant.

## 3. Results

### 3.1. The Effects of ADM, H_2_O_2_, and GSH on the Viability of MCF-7 and MCF-7/ADM Cells

To optimize the concentrations of different treatments for conducting long-term incubation experiments, MCF-7 cells were treated with different concentrations of ADM, H_2_O_2_, or GSH. Two days after the treatment, the cell viability was measured by SRB assay; and our results (Figure 2) showed that ADM (≥5 *μ*M), H_2_O_2_ (≥100 *μ*M), and GSH (≥10 mM) significantly inhibited the viability in MCF-7 cells. Then, lower concentrations were applied for long-term incubation (two weeks and one month, data not shown). However, cells could not survive for more than one month when they were continuously treated with H_2_O_2_ or ADM with a concentration higher than 1 *μ*M. GSH at concentration of 1 mM did not show significant cytotoxicity, but the iROS was almost abolished when cells were treated with 1 mM GSH for 48 h. Based on these results, concentrations of 0.001, 0.01, and 0.1 *μ*M for H_2_O_2_, concentrations of 0.001, 0.01, and 0.1 mM for GSH, and concentration of 0.1 *μ*M for ADM were used in the following studies.

To optimize the treatment duration, drug administration was performed for more than 6 months, and MDR was evaluated by using MTT assay every 2 weeks. MDR was found gradually increased during the first 20 weeks, and then the MDR was maintained in a steady level without further enhancement (data not shown). Therefore, 20 weeks were selected as the optimal treatment duration in the following studies.

### 3.2. MDR Was Induced by 20-Week Exposure of H_2_O_2_, GSH, and ADM in MCF-7 Cells

After 20-week incubation with different treatments, MDR to ADM or taxol was determined by MTT and SBR assays in MCF-7 cells (Figure 3). Long-term (20 weeks) treatment of H_2_O_2_ and GSH both increased the IC_50_ values in a concentration-dependent manner when compared to control. Furthermore, in the MCF-7 cells treated with ADM (0.1 *μ*M) for 20 weeks, the IC_50_ values were also significantly increased compared to control, while cotreatment with GSH (2 mM) significantly decreased the IC_50_ values when compared with cells treated with ADM alone. Since 2 mM GSH can almost eliminate the ADM-induced ROS, the result further proved the close relationship between ROS and MDR generations.

### 3.3. The Drug Accumulation Ability of MCF-7 Cells Was Decreased by Long-Term Treatment of H_2_O_2_ and GSH

**Figure 4:**
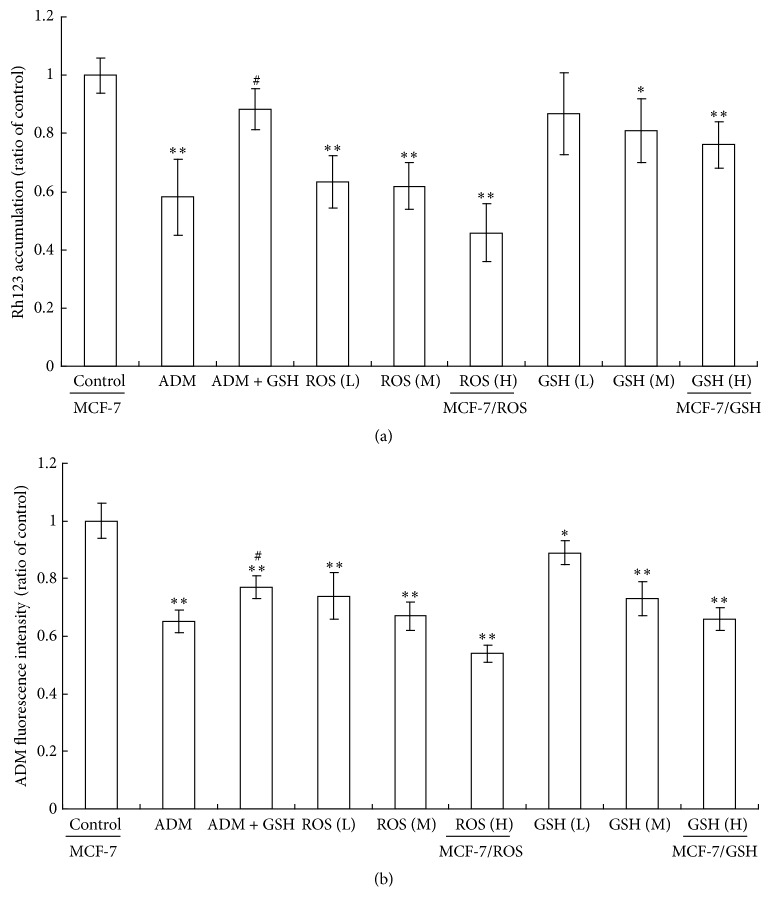
The accumulation ability of Rh123 (a) and ADM (b) was decreased by long-term treatment of H_2_O_2_ and GSH. MCF-7 cells were treated by replacing the culture medium every other day for 20 weeks. The accumulation ability was determined by flow cytometry. Group design and statistical analysis refer to Figure 3.

Further, we evaluated the effects of different treatments on drug accumulation ability as measured by flow cytometry. Our results showed that ADM long-term treatment caused a decrease in both Rh123 and ADM accumulations when compared to control, while cotreatment with GSH antagonized the effect of ADM on drug accumulation. Long-term treatment of H_2_O_2_ or GSH both concentration-dependently decreased drug accumulation in MCF-7 cells, especially in cells incubated with 0.1 *μ*mol/L H_2_O_2_ (Figure 4). Since Rh123 and ADM are classical substrates of MDR transporters like P-gp and MRP1, the depressed drug accumulation ability suggested the generation of MDR.

### 3.4. The Effects of Different Treatments on the Cell Viability and Proliferative Ability

**Figure 5:**
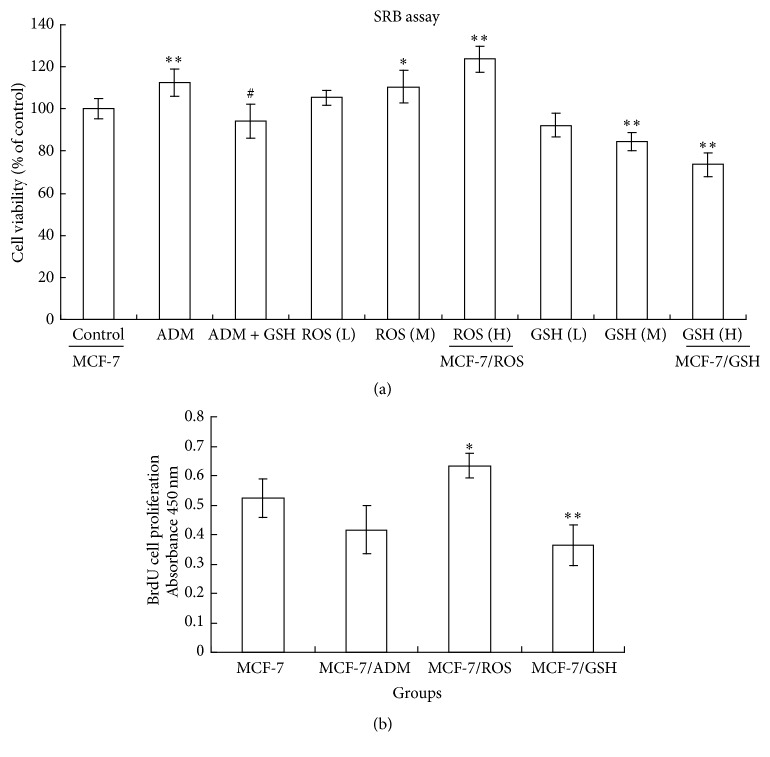
Effects of different long-term treatments on the cell viability and of proliferative ability in MCF-7. (a) Cell viability was determined by SRB assay. (b) Proliferative ability of MCF-7, MCF-7/ADM, MCF-7/ROS, and MCF-7/GSH cells was analyzed by BrdU incorporation assay. MCF-7 cells were treated by replacing the culture medium every other day for 20 weeks. Group design and statistical analysis refer to Figure 3.

The results of SBR assay (Figure 5(a)) showed that long-term ADM treatment increased the viability of MCF-7 cells. But cotreatment with GSH abolished the effects of ADM on cell viability. Consistently, the long-term treatment of H_2_O_2_ also increased the viability of MCF-7 cells in a concentration-dependent manner. However, the long-term treatment of GSH had an opposite effect that the cell viability was inhibited. In the BrdU incorporation assay (Figure 5(b)), compared with the control MCF-7 cells, the proliferative ability was higher in MCF-7/ROS cells. On the contrary, the proliferative ability in MCF-7/ADM and MCF-7/GSH cells was much lower than that of MCF-7 cells. These findings indicated that the cell viability and proliferation of MDR cells varied depending on the cultivation agents.

### 3.5. Long-Term Treatment of H_2_O_2_ and GSH Caused Decreased iROS

**Figure 6:**
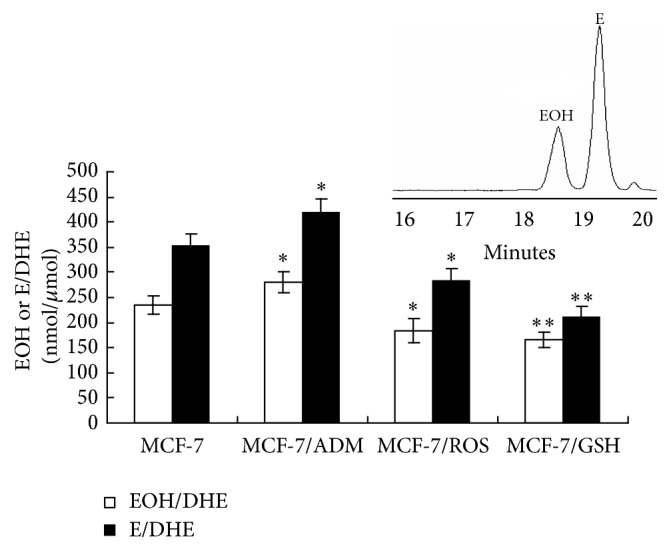
The iROS level in MCF-7, MCF-7/ADM, MCF-7/ROS, and MCF-7/GSH cells. MCF-7 and MCF-7/ADM cells were obtained from American Type Culture Collection (ATCC, Manassas, VA, USA) and KeyGen Biotech (Nanjing, China). MCF-7/ROS and MCF-7/GSH cells were MCF-7 cells, respectively, treated with 0.1 *μ*M H_2_O_2_ and 0.1 mM GSH every other day for successive 20 weeks. Cells were maintained in PBS and incubated with 50 *μ*M DHE for 30 min and then harvested to analyze DHE-derived products by HPLC. Inset, representative chromatogram profile of EOH and E separation in cell extract. Data represents the mean ± SD, *n* = 3. Significant differences relative to MCF-7 cells were indicated as ^*∗*^ *P* < 0.05 and ^*∗∗*^ *P* < 0.01.

EOH is generated when DHE is oxidized by anion superoxide, while ethidium production is associated with heme proteins levels and peroxidase activity. Therefore, as shown in Figure 6, MCF-7/ADM cells have higher iROS levels than MCF-7 cells, which is similar to the results of DCF stain (supplementary Figure 1 in Supplementary Material available online at http://dx.doi.org/10.1155/2016/7053451). Results also showed that both H_2_O_2_ and GSH long-term treatments decreased the levels of iROS when compared to MCF-7 cells. We speculate that the difference of iROS between MCF-7/ADM cells and our models may be due to the different modeling methods. In addition, although HE treatment in ROS assay was reported to be toxic by gradual depolarization of mitochondrial membrane in K562 cells [20], we did not observe any toxicity during the assay in this study.

### 3.6. Long-Term Treatment of H_2_O_2_ and GSH Caused Alterations on Intracellular Antioxidants

**Figure 7:**
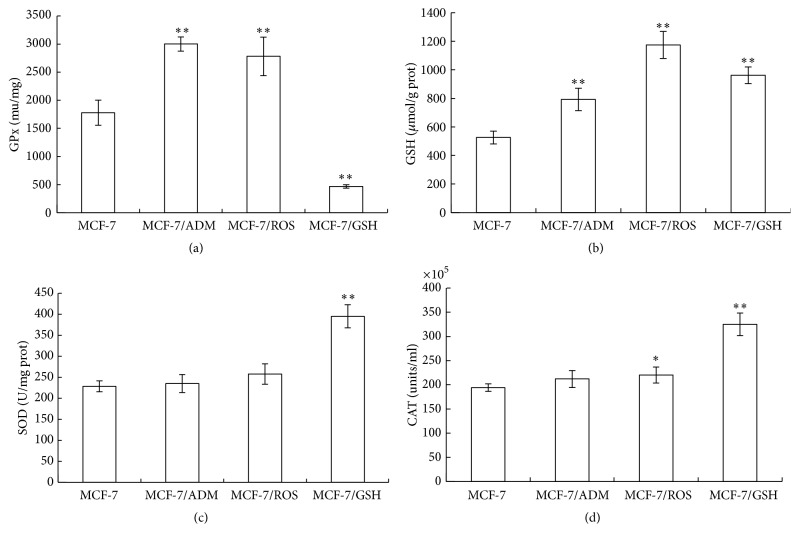
The differences of GPx (a), GSH (b), SOD (c), and CAT (d) in MCF-7, MCF-7/ADM, MCF-7/ROS, and MCF-7/GSH cells. Group design and statistical analysis refer to Figure 6.

As shown in Figure 7, compared with the control MCF-7 cells, the GPx, SOD, and CAT activities, as well as the GSH content, were higher in MCF-7/ROS cells. MCF-7/GSH cells only had significantly increased SOD, CAT activity, and GSH content. The GPx activity in MCF-7/GSH cells was lower than that of control cells. In addition, MCF-7/ADM cells only had significant higher GPx and GSH compared with MCF-7 cells. Although different modeling methods caused various features of intracellular antioxidants, these findings further revealed the close relationship between ROS-induced MDR and the alterations of intracellular antioxidants.

### 3.7. ROS-Induced MDR in MCF-7 Cells Was Correlated with Upregulation of Drug Transporters

**Figure 8:**
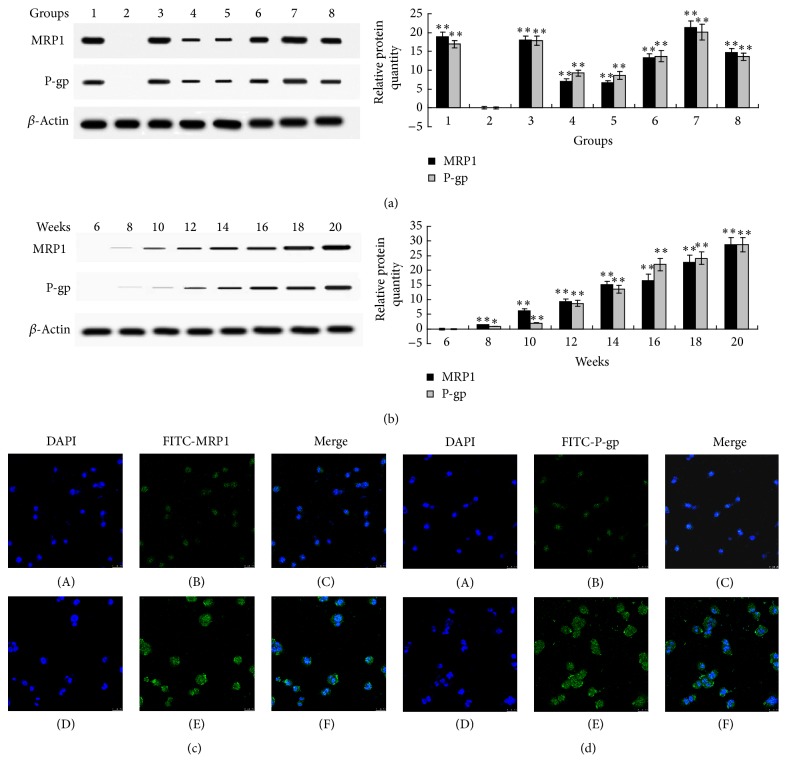
ROS-induced MDR in MCF-7 cells was correlated with upregulation of MRP1 and P-gp. (a) MRP1 and P-gp protein expression levels were determined by western blot assay. 1, MCF-7/ADM cells; 2, MCF-7 cells (control); 3, MCF-7 cells treated with 0.1 *μ*M ADM; 4, MCF-7 cells treated with 0.1 *μ*M ADM + 2 mM GSH; 5, MCF-7 cells treated with 0.001 *μ*M H_2_O_2_; 6, MCF-7 cells treated with 0.01 *μ*M H_2_O_2_; 7, MCF-7 cells treated with 0.1 *μ*M H_2_O_2_; 8, MCF-7 cells treated with 0.1 mM GSH; the treatment duration was 20 weeks. (b) Effects of H_2_O_2_ (0.1 *μ*M) treatment on protein expression levels of MRP1 and P-gp were determined by western blot. Samples were obtained at the 6th, 8th, 10th, 12th, 14th, 16th, 18th, and 20th week of H_2_O_2_ incubation. (c) MRP1 and (d) P-gp were labeled by double fluorescence staining using DAPI and FITC-labeled antibodies. (A)–(C) MCF-7 cells; (D)–(F) MCF-7/ROS cells (MCF-7 cells treated with 0.1 *μ*M H_2_O_2_ for 20 weeks). The confocal fluorescence image was captured at magnification of 400x, scale bar = 25 *μ*m. Data represents the mean ± SD, *n* = 3. Significant differences relative to control group were indicated as ^*∗*^ *P* < 0.05 and ^*∗∗*^ *P* < 0.01.

Western blot results (Figure 8) showed that both MCF-7/ADM and MCF-7 cells which received long-term 0.1 *μ*M ADM treatment had higher protein expression levels of MRP1 and P-gp than MCF-7 cells. Cotreatment with GSH prevented the increase in MRP1 and P-gp protein levels in MCF-cells which received ADM treatment. Further, H_2_O_2_ increased the protein expression levels of MRP1 and P-gp in a concentration-dependent and time-dependent manner. GSH long-term treatment also caused an increase in the protein expression levels of MRP1 and P-gp. Confocal microscopy results further confirmed that H_2_O_2_ induced upregulation of MRP1 and P-gp on the cell membrane of MCF-7 cells (Figures 8(c) and 8(d)).

### 3.8. ROS-Induced Expression of MDR-Related Factors in MCF-7 Cells

**Figure 9:**
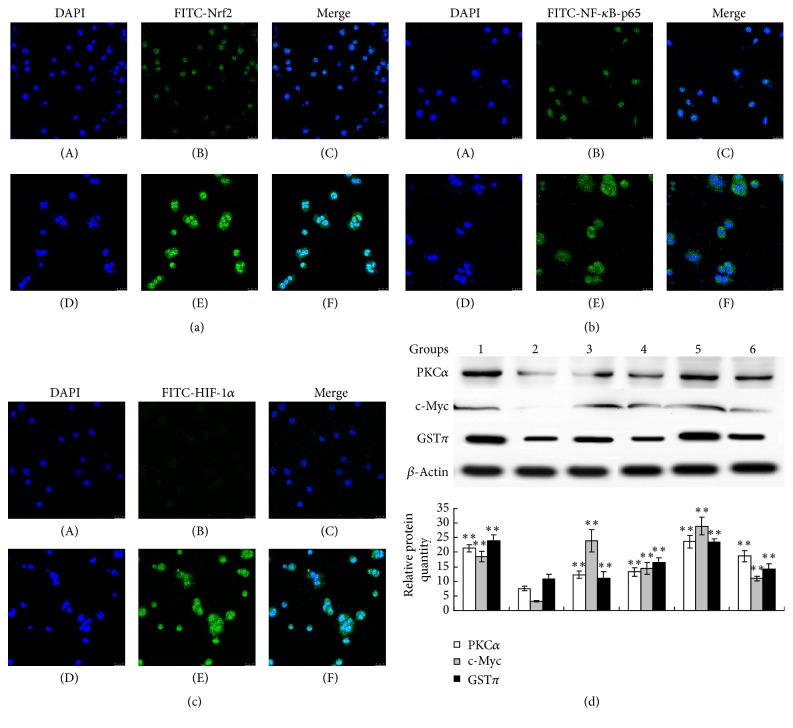
ROS-induced expression of MDR-related factors in MCF-7 cells. (a) Nrf2, (b) NF-*κ*B-p65, and (c) HIF-1*α* were labeled by double fluorescence staining using DAPI and FITC-labeled antibodies. (A)–(C) MCF-7 cells; (D)–(F) MCF-7/ROS cells (MCF-7 cells treated with 0.1 *μ*M H_2_O_2 _for 20 weeks). The confocal fluorescence image was captured at magnification of 400x, scale bar = 25 *μ*m. (d) The expression levels of PKC*α*, c-Myc, and GST*π* were analyzed by western blot. 1, MCF-7/ADM cells; 2, MCF-7 cells (control); 3, MCF-7 cells treated with 0.1 *μ*M ADM; 4, MCF-7 cells treated with 0.1 *μ*M ADM + 2 mM GSH; 5, MCF-7 cells treated with 0.1 *μ*M H_2_O_2_; 6, MCF-7 cells treated with 0.1 mM GSH; the treatment duration was 20 weeks. Data represents the mean ± SD, *n* = 3, and significant differences relative to control group were indicated as ^*∗*^ *P* < 0.05 and ^*∗∗*^ *P* < 0.01.

In order to further elucidate the underlying mechanisms of ROS-induced MDR, immunofluorescence staining was performed to examine several transcriptional factors in close relationship with oxidative stress, including Nrf2, NF-*κ*B-p65, and HIF-1*α*, which were also widely reported as regulators of efflux transporter like P-gp and MRP1 [21–24]. Our results showed that Nrf2, NF-*κ*B-p65, and HIF-1*α* were found to be highly expressed in MCF-7/ROS cells compared to control MCF-7 cells. And the increased Nrf2 and HIF-1*α*, in particular, concentrated in the nuclei suggesting them in activation (Figures 9(a), 9(b), and 9(c)). Moreover, it has been well established that the overexpressions of PKC*α*, c-Myc, and GST*π*, which, respectively, signify strong ability of ROS scavenging and P-gp overexpression [25, 26], apoptosis resistance and high proliferation [27], and overactivation of cellular detoxification [28], were classic mechanisms of MDR. Therefore, western blot was performed to determine these protein levels in MCF-7/ADM cells and MCF-7 cells with different treatments. Results showed that protein levels of PKC*α*, c-Myc, and GST*π* in MCF-7/ADM cells were significantly higher than that in control MCF-7 cells (Figure 9(d)). Notably, the long-term treatment with 0.1 *μ*M ADM, 0.1 *μ*M H_2_O_2_, and 0.1 mM GSH all increased the protein levels of PKC*α*, c-Myc, and GST*π* (Figure 9(d)). Cotreatment with 2 mM GSH partially attenuated the effects of 0.1 *μ*M ADM on the protein levels of PKC*α*, c-Myc, and GST*π* in MCF-7 cells.

### 3.9. PI3K/Akt, Nrf2, and HIF-1*α* Signaling Pathways Involved in ROS-Induced MDR in MCF-7 Cells

**Figure 10:**
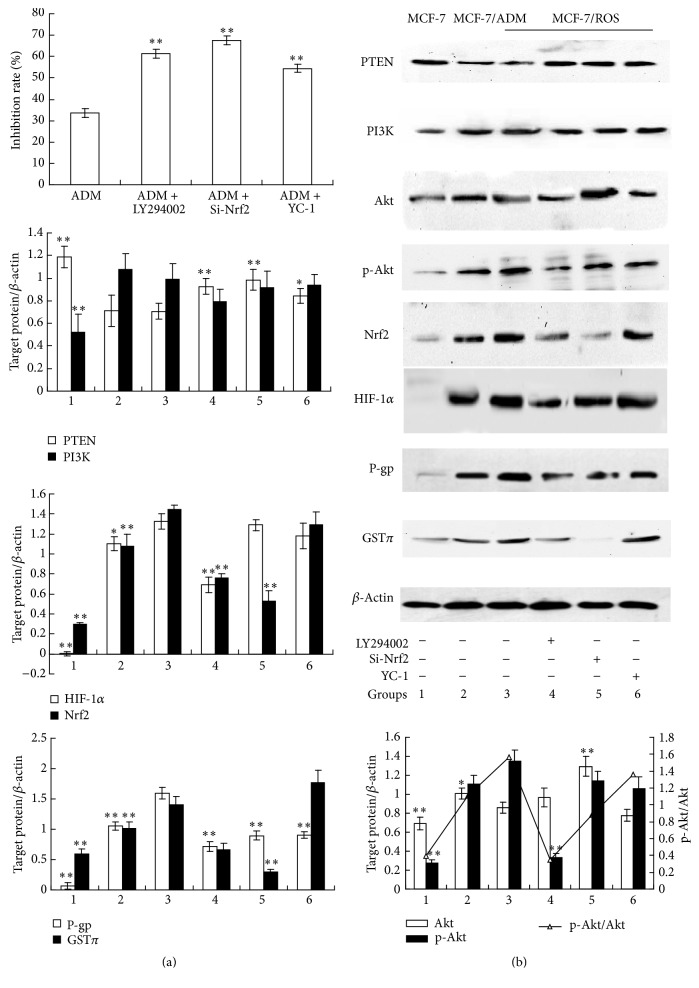
PI3K/Akt, Nrf2, and HIF-1*α* signaling pathways involved in ROS-induced MDR in MCF-7 cells. (a) PI3K/Akt, Nrf2, and HIF-1*α* inhibition increased the inhibition rate of MCF-7/ROS cells (MCF-7 cells were treated with 0.1 *μ*M H_2_O_2_ for 20 weeks) by ADM. MCF-7/ROS cells were treated with 125 *μ*M ADM, 125 *μ*M ADM + 10 *μ*M LY294002, 125 *μ*M ADM + 5 *μ*M YC-1, or 125 *μ*M ADM + Si-Nrf2 for 48 h, and then the inhibition rate was determined by MTT assay. Data represents the mean ± SD, *n* = 3, and significant differences relative to MCF-7/ROS cells which only received 125 *μ*M ADM were indicated as ^*∗∗*^ *P* < 0.01. (b) Protein levels of PTEN, PI3K, Akt, p-Akt, Nrf2, HIF-1*α*, P-gp, and GST*π* in MCF-7 cells (group 1) and MCF-7/ADM cells (group 2) and in MCF-7/ROS cells which received 10 *μ*M LY294002 (group 4), Si-Nrf2 (group 5), or 5 *μ*M YC-1 (group 6) treatment for 48 h were determined by western blot. Data represents the mean ± SD, *n* = 3, and significant differences relative to control group (MCF-7/ROS cells, group 3) were indicated as ^*∗*^ *P* < 0.05 and ^*∗∗*^ *P* < 0.01.

Since the activations of Nrf2 and HIF-1*α* were found in MCF-7/ROS cells, further study was to determine whether they participated in the regulation on MDR and to reveal their possible upstream pathway and downstream targets. To do this, Si-Nrf2 and specific HIF-1*α* inhibitor YC-1 were applied to detect the inhibition rate induced by 125 *μ*M ADM on MCF-7/ROS cells via MTT assay. The results of the PCR experiments proved that the mRNA of Nrf2 in MCF-7/ROS cells was decreased by 84.4 ± 7.6% after siRNA treatment for 48 h. In addition, nontargeting siRNA constructs were used as control in the Si-Nrf2 assays (shown in supplementary Figure 2). As shown in Figure 10(a), the inhibition rate of 125 *μ*M ADM was 33.70 ± 2.02%. When the cells were treated with Si-Nrf2 or YC-1, the inhibition rate was significantly increased to 67.43 ± 2.01% and 54.42 ± 1.8%, respectively. Furthermore, the protein level of P-gp was significantly decreased in the presence of Si-Nrf2 and YC-1 while the protein level of GST*π* was reduced by Si-Nrf2. Since PI3K/Akt pathway was usually recognized as an activator in the Nrf2-mediated antioxidant response [29] and an enhancer in the protein translation of HIF-1*α* [30], we further investigated whether the PI3K/Akt pathway participated in the transduction of the Nrf2 and HIF-1*α* effects on MCF-7/ROS cells. As shown in Figure 10, specific PI3K inhibitor LY294002 increased ADM-induced inhibition rate to 61.31 ± 2.11% and significantly depressed the protein levels of Nrf2 and HIF-1*α*, as well as their downstream targets P-gp and GST*π*. Together, these data indicated that the long-term treatment of H_2_O_2_ contributed to MDR with high expressions of P-gp and GST*π* in MCF-7/ROS cells via activations of Nrf2 and HIF-1*α* which were regulated by PI3K/Akt pathway.

## 4. Discussion

MDR is a major obstacle to the successful cancer chemotherapy, and it involves various mechanisms, mainly including overexpressed efflux transporters, overactivated detoxification system, and imbalanced apoptosis/proliferation [27]. Since functional crosstalk in the signaling pathways between various phenotypes of MDR has been reported in many MDR cancers [31], it is important for us to identify the common mediator in the signaling pathways among different phenotypes of MDR, which may help us to pinpoint critical targets for a better control of MDR during cancer chemotherapy.

The importance of ROS in MDR has been emphasized in various studies, but there is no unified conclusion in the relationship between ROS and MDR. Our studies have demonstrated the differences in the iROS levels between MDR cells and their parental sensitive cells, in which the level of iROS in MCF-7/ADM cells was significantly higher than that in MCF-7 cells. It is consistent with the findings showing a higher level of iROS in MDR cells of KBv200 and LoVo DX [32, 33]. However, several other studies demonstrated that the levels of iROS in MDR cells from ovarian carcinoma, Lucena cells, and HOB1/VCR cells were reported lower than that in sensitive ones [34–36]. On the other hand, the effects of ROS on MDR were also reported to be inconsistent among different studies. For instance, Eidus et al. revealed that MDR was enhanced by ROS activated PKC in HEp-2 cells [11], and the increase in ROS levels also caused an upregulation of P-gp in various types of cells, including cancer cells and brain microvessel endothelial cells [37, 38]. Moreover, it has been well established that ROS enhancement could lead to drug resistance in bacteria [8]. On the contrary, our previous study revealed that an anthraquinone analogue downregulated P-glycoprotein expression in K562/DOX cells via generation of ROS [12], and Emel'yanov et al. also demonstrated that H_2_O_2_ reversed MDR in P388VR cells in a time/concentration-dependent manner within 1 h [39]. In addition, MDR could be circumvented by various drugs via ROS generation [40]. To the best of our knowledge, clinically, generation of MDR may be due to the prolonged and repeated chemotherapy, that is, more than several months, and iROS level in one cell line is the result of long-term dynamic redox equilibrium. However, the above conclusions were drawn from the studies based on short-term treatments. In order to mimic the clinical condition in the development of MDR, long-term intervention with oxidant/antioxidant was employed in our models, by which we investigate the effects of long-term H_2_O_2_/GSH treatment on MDR in MCF-7 cells to reveal the mechanisms linking oxidant levels with the establishment of MDR.

Interestingly, our results showed that long-term incubation of both H_2_O_2_ and GSH led to MDR, which is consistent with Korystov's findings showing that bidirectional regulation of ROS led to MDR via activation of different signaling pathways [41]. Since the dual effects of ROS on cell proliferation and apoptosis have been well documented, it is quite likely that ROS may also play dual roles in MDR in cancer cells. Importantly, the level of iROS was all found depressed with the enhancement of intracellular antioxidants in our model. It was easy to understand the decrease of iROS by GSH treatment; as for the decrease of iROS by H_2_O_2_ treatment, we proposed the idea that oxidants in low concentrations may activate detoxification system to increase cellular adaptation to oxidative stress. Therefore, in our study, H_2_O_2_ treatment and GSH treatment both caused a decrease in iROS levels with MDR generation in MCF-7 cells, which suggests the importance of ROS in the generation of MDR. However, it is still hard to find an appealing explanation for why MCF-7/ADM cells had higher levels of iROS than MCF-7 cells. One possible reason may be that the iROS level varies in different cell origins, types, cultural methods, and so forth, and so does the cellular response to ROS, as it is reported that upregulation of P-pg by reduction of ROS levels was only observed in HepG2 cells but not in MCF-7, A549, A431, HeLa, and Hvr100-6 cell lines [42, 43].

**Figure 11:**
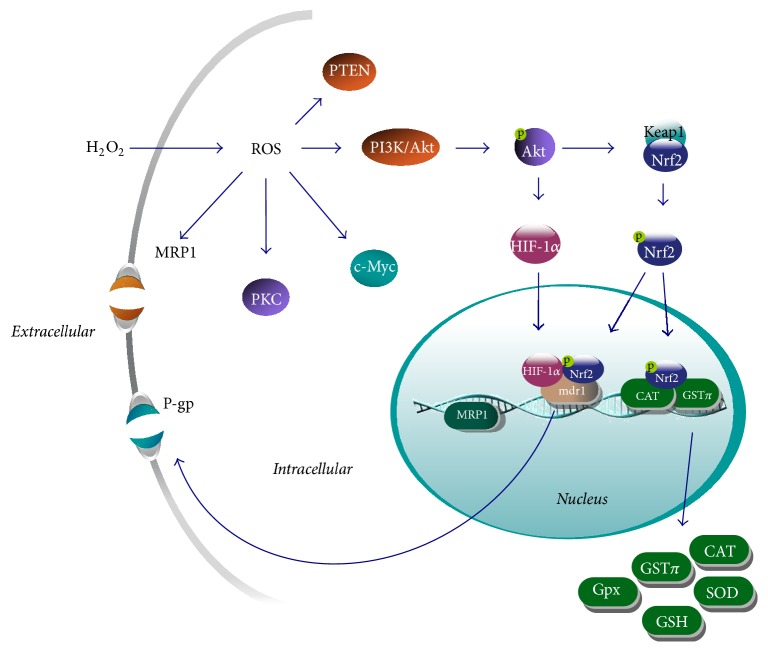
Proposed signaling pathways involved in ROS-induced MDR in MCF-7 cells.

Given the successful establishment of our MDR cell model, the detailed mechanism of ROS-induced MDR was further revealed. Firstly, overexpressions of functional P-gp and MRP1, which are classic efflux mechanisms of anticancer drugs and correlate broadly with negative treatment response [44], were found in MCF-7 cells after long-term treatment with both H_2_O_2_ and GSH. Secondly, the transcriptional factors of efflux transporter, such as Nrf2, NF-*κ*B, and HIF-1*α* [21–24], were also upregulated in MCF-7/ROS cells. Importantly, the elevated levels of Nrf2 and HIF-1*α* were mainly localized in the nuclei of MCF-7/ROS cells, which suggested them in activation. There is overwhelming evidence that molecular events leading to MDR are regulated by the inducible Nrf2-linked pathway, a key switch-on mechanism for upregulation of endogenous antioxidant enzymes and detoxifying systems [22]. In line with this, our results also revealed that transient silence of Nrf2 led to the sharp dropping of GST*π* expression, suggesting that ROS-induced antioxidant system was mainly through the activation of Nrf2 signal pathway in MCF-7/ROS cells. Although HIF-1*α* was usually regarded as a mediator to hypoxia [45], its upregulation in MCF-7/ROS cells suggests the important role of HIF-1*α* in oxidative response and in MDR, as shown earlier [46]. However, the distribution of NF-*κ*B was mainly found in cytoplasm. Since NF-*κ*B can also be directly regulated by ROS bidirectionally [47], we proposed that its expression and distribution might be a complex consequence of functional linage among different signaling pathways. Thirdly, our result revealed that Nrf2/HIF-1*α* overexpressed P-gp and GST*π* are mediated at least partially by PI3K/Akt pathway. The related signal cascades in ROS-induced MDR were analyzed by using the inhibitor of PI3K and HIF-1*α*, as well as siRNA for Nrf2 on MCF-7/ROS cells. It is well known that overactivated PI3K/Akt pathway is a common feature of solid tumors [29]. There is now accumulated evidence that high levels of PI3K and overactivated downstream target Akt can activate the Nrf2-mediated antioxidant response, whereas PTEN can inhibit it [30]. Furthermore, PI3K can upregulate the HIF-1*α* protein translation [48]. Studies have been also showing that the induction of the Nrf2 pathway augments HIF-1*α* signaling [49]. Consistent with these reports, our results found that PI3K specific inhibitor LY294002 significantly restored the sensitivity of MCF-7/ROS cells to ADM. More importantly, HIF-1*α* and Nrf2 were manifested as the downstream targets of overactivated PI3K/Akt pathway, while they were both responsible for the overexpression of P-gp in MCF-7/ROS cells. However, since LY294002 did not totally abolish the MDR and the expression of P-gp, it is possible that other signals participate in the MDR of MCF-7/ROS cells. Fourthly, PKC*α*, c-Myc, and GST*π* were found significantly augmented in both MCF-7/ROS and MCF-7/GSH cells. Several reports suggested that increased levels of PKC isoforms, mainly for PKC*α*, were found in MDR cells linking with activated P-gp [26]. C-Myc, a prosurvival/antiapoptosis factor, has recently been reported to participate, at least partly, in MDR to some types of cancers [27]. It was established that c-Myc is responsible for directing and coordinating the transcription of MDR transporters in leukemia and human colon carcinoma [50]. Our previous study also showed that c-Myc was strongly involved in the resistance of MCF-7/ADM cells [27]. In addition, GST-*π* is a multifunctional enzyme that plays a critical role in cellular detoxification. It is considered to be associated with the efflux of many antitumor drugs through ATP-binding cassette transporters [28]. Accordingly, overexpressions of c-Myc, GST*π*, and PKC*α* were also important mechanisms of MDR in our models. Furthermore, the treatment of GSH in ADM group greatly eliminated ADM-induced MDR and the expressions of corresponding factors. Therefore, our studies suggest that ROS can be an independent inducer of MDR and a key factor in the crosstalk of various mechanisms of MDR. Together, the possible cascades of those signal pathways were shown in Figure 11, and other signaling pathways in ROS-induced MDR are still yet to be investigated in further studies.

In conclusion, the levels of iROS were different in MCF-7 and MCF-7/ADM cells. Long-term bidirectional modulation of ROS led to MDR in MCF-7 cells. In the present study, we demonstrated that ROS act as key inducers in MDR and participate in various MDR phenotypes, including overexpressed efflux transporters (MRP1 and P-gp), overactivated antioxidation system (Nrf2 and GST*π*), and apoptosis resistance (HIF-1*α*, c-Myc, and PKC*α*). Moreover, a novel MDR model (MCF-7/ROS) was developed in this study by long-term incubation of H_2_O_2_, which was readily prepared with low cost and high proliferation. Our study suggests that ROS play critical roles in the development of MDR in breast cancer cells.

## Supplementary Material

Flow cytometry results showed that MCF-7/ADM cells from different sources have higher iROS levels than MCF-7 cells. The ratio was 1.59±0.09 folds (supplementary Figure 1 A) for cells from China Pharmaceutical University (Nanjing, China) and 2.77±0.21 (supplementary Figure 1 B) for cells from Keygen Biotech (Nanjing, China). Moreover, our confocal microscopy results further demonstrated that there was an increase in the fluorescence intensity of DCF, which was evenly distributed all over the MCF-7/ADM cells (supplementary Figure 1 C).
