# Supplementary figures and images for: Long-Term Alteration of Reactive Oxygen Species Led to Multidrug Resistance in MCF-7 Cells
Source: Oxid Med Cell Longev. 2016 Dec 12;2016:7053451. doi: 10.1155/2016/7053451 (PMC5183793; doi:10.1155/2016/7053451)

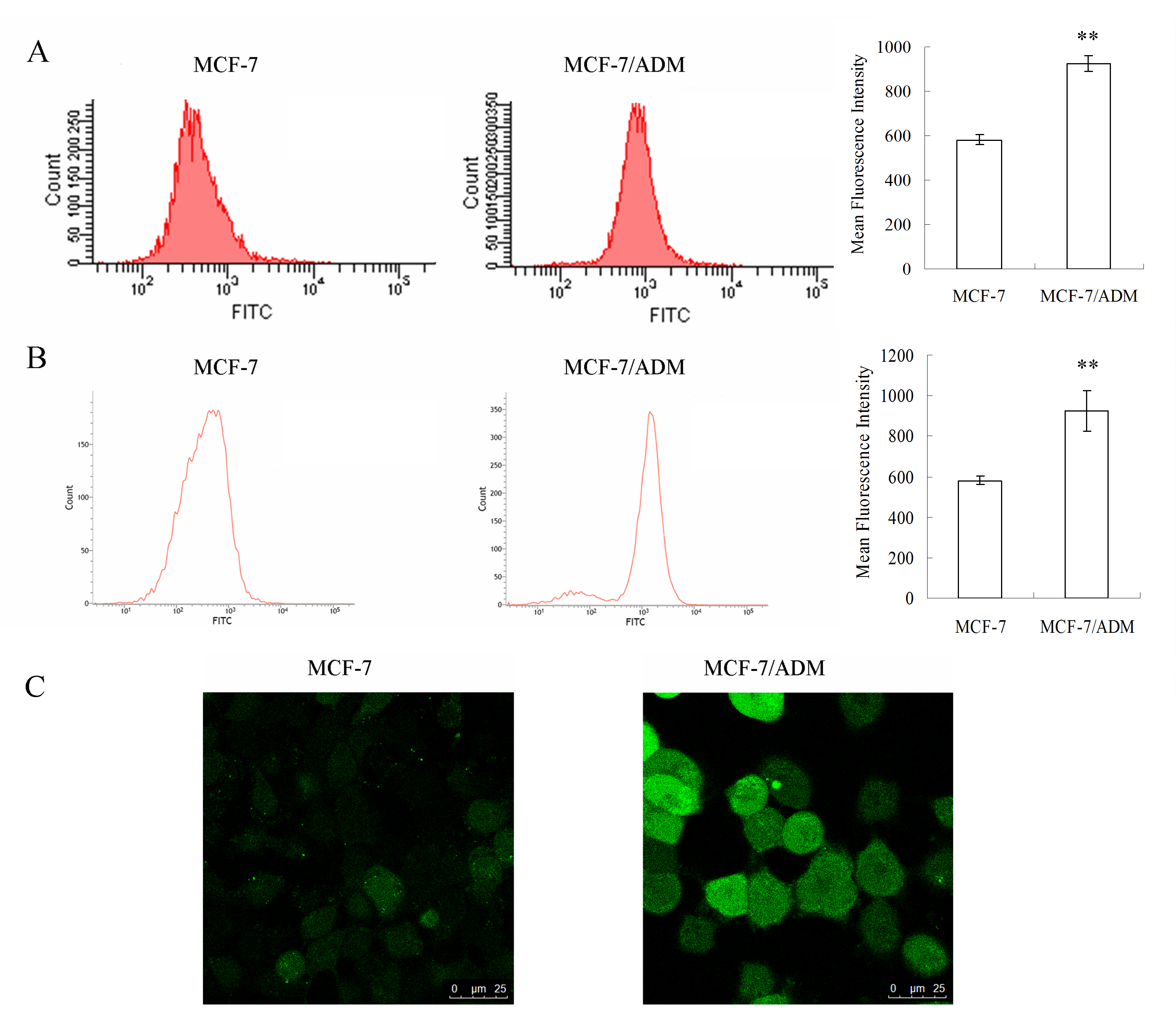

Supplement: Supplementary file 1 — Flow cytometry results showed that MCF-7/ADM cells from different sources have higher iROS levels than MCF-7 cells. The ratio was 1.59±0.09 folds (supplementary Figure 1 A) for cells from China Pharmaceutical University (Nanjing, China) and 2.77±0.21 (supplementary Figure 1 B) for cells from Keygen Biotech (Nanjing, China). Moreover, our confocal microscopy results further demonstrated that there was an increase in the fluorescence intensity of DCF, which was evenly distributed all over the MCF-7/ADM cells (supplementary Figure 1 C). [file 7053451.f1.zip › Supplementary material/Supplementary Fig.1_OMCL_1795544.tif]

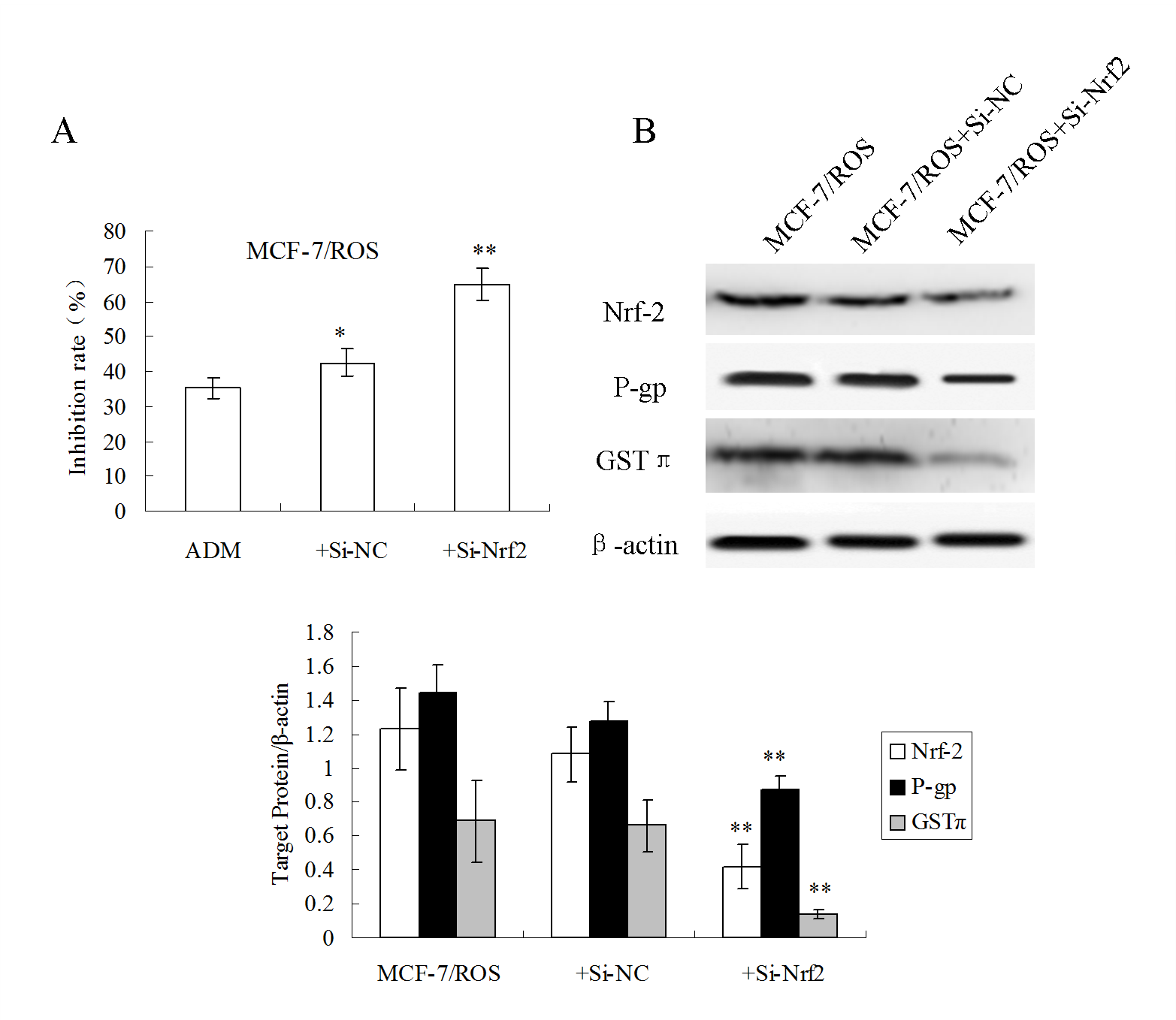

Supplement: Supplementary file 1 — Flow cytometry results showed that MCF-7/ADM cells from different sources have higher iROS levels than MCF-7 cells. The ratio was 1.59±0.09 folds (supplementary Figure 1 A) for cells from China Pharmaceutical University (Nanjing, China) and 2.77±0.21 (supplementary Figure 1 B) for cells from Keygen Biotech (Nanjing, China). Moreover, our confocal microscopy results further demonstrated that there was an increase in the fluorescence intensity of DCF, which was evenly distributed all over the MCF-7/ADM cells (supplementary Figure 1 C). [file 7053451.f1.zip › Supplementary material/Supplementary Fig.2_OMCL_1795545.tif]
